# Do 'good values' lead to 'good' health-behaviours? Longitudinal associations between young people's values and later substance-use

**DOI:** 10.1186/1471-2458-10-165

**Published:** 2010-03-26

**Authors:** Robert Young, Patrick West

**Affiliations:** 1 MRC Social and Public Health Sciences Unit, University of Glasgow, 4 Lilybank Gardens, Glasgow G12 8RZ, UK

## Abstract

**Background:**

Past studies have linked certain values (traditional vs. individualistic) with adolescent substance-use. The aims of this study are to replicate cross-sectional research linking values and adolescent substance-use and to determine if such values predict future substance-use.

**Methods:**

A longitudinal school-based survey of 2196 young people (age 15) followed up in early adulthood (age 18/19). Participants provided data about their beliefs and values at age 15, and their substance-use (smoking, alcohol and drug-use) at ages 15 and 18/19. In addition data were collected about their social background (gender, risk-taking, deprivation, religion, etc).

**Results:**

Cross-sectionally, young people with anti-authority values were more likely to use various substances, e.g. 17-67% more likely to regularly smoke (daily), drink (most days), or use drugs (weekly) for each SD above typical levels. Adjusting for social background, associations were not substantially attenuated. However in the prospective analysis, adjusting for both background and substance-use at age 15, only two (anti-authoritarian and work ethic) values were (marginally) associated with substance-use at age 18/19.

**Conclusions:**

While we replicated results found in prior cross-sectional studies, evidence from this study does not support the argument that holding certain 'pro-social' or 'good' values substantively protects against later substance-use and challenges the likely effectiveness of values-based interventions in relation to later substance-use.

## Background

Character or values education is the policy of using the school curriculum to influence young people's values, typically promoting traditional and citizenship values. It is a topic which provokes controversy in its own right [1-3], even more so when linked to risky or health-compromising behaviours such as substance-use [4]. Proponents argue that because values are strong cognitive, emotionally significant guiding and organising principles in an individual's life, they substantially shape both their current and future health behaviours. However, surprisingly little research has ventured beyond simple cross-sectional analyses between young people's values and health-behaviours. Accordingly, we examine the longitudinal association between values and substance-use (tobacco, alcohol and illegal drug-use), a topic of considerable current public health concern.

Before reviewing the limited evidence linking values and substance-use, we briefly outline the controversies surrounding values or character education, define what 'personal or human values' are and how such values are measured.

### Controversies in values education

Proponents of values education make strong claims for its effectiveness, as for example in Lickona's '*Combating Violence With Values: The Character Education Solution*' [4]. These claims are contested equally strongly by opponents such as Law [1]. Proponents (typically with religious affiliations) argue that promoting basic values such as equality, citizenship and obedience is not only intrinsically worthwhile but also bestows positive health [4]. Representatives of the liberal/secular position argue for the promotion of values such as freedom of expression or action, while acknowledging they often conflict with traditional values, e.g. blasphemy [1] (the challenge by free speech advocates towards the new 2010 Irish blasphemy law is a recent European example: http://news.bbc.co.uk/1/hi/world/europe/8437460.stm).

Much of the debate originates in America, with ex-president George W. Bush among the many advocates of character education [1]. During his period of office, the US Department of Education allocated approximately $25,000,000 each year from 2002-6 to developing school character programs http://www.ed.gov/programs/charactered/funding.html. However, the topic is relevant beyond the American context since virtually all western educational systems incorporate some form of values education. For example, in the UK much of what could be termed values education (particularly citizenship) is covered in the Personal and Social Education curriculum [5].

The debate is largely conducted in the absence of any reliable evidence, with a recent Australian government review summarising the evidence base thus: "*Values education can be described, according to the literature review, as a subject about which much has been written but little is known*." (page 33) [6]. Against this background, arguments about what constitutes the most appropriate values to promote, or which values lead to good health, remain unsubstantiated.

### Values

**Figure 1:**
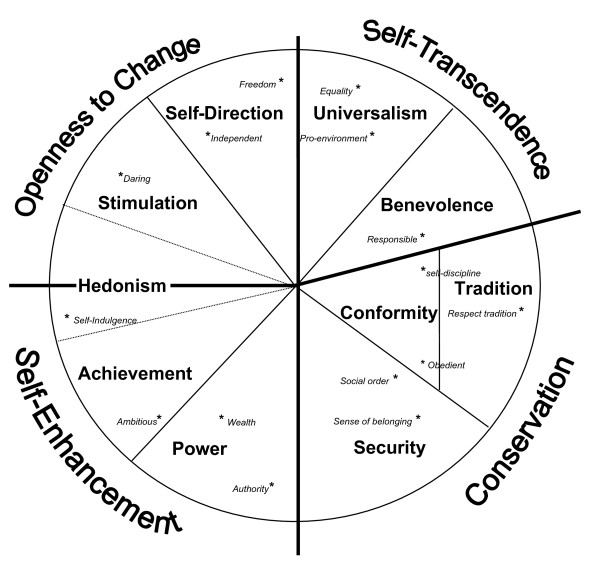
**Theoretical model of the Structure of Values and approximate location of selected representative items derived from empirical studies (smallest space analysis)**.

If there is a lack of empirical evidence in relation to values education, the opposite is true for the psychological study of values. There is extensive cross-cultural agreement regarding the measurement and structure of values, which are broadly defined as *"desirable goals, varying in importance, that serve as guiding principles in people's lives." *[7]. The additional file 1 outlines one leading framework by Schwartz [8,9]. Briefly, Schwartz defines ten generic values: benevolence, universalism, self-direction, stimulation, hedonism, achievement, power, security, conformity, and tradition. For example, conformity is defined as "*restraint of actions, inclinations, and impulses likely to upset or harm others and violate social expectations or norms*" [10] and is measured by opinions such as the importance of honoring elders and obedience. Further, the ten values can be collapsed into two perpendicular poles, each quadrant representing typical groupings (openness-to-change vs. conservation and self-transcendence vs. self-enhancement; figure 1 and additional file 1). In our study, these two poles and four groups of values can be summarised as traditional (Schwartz's conservation) vs. self-direction (Schwartz's openness-to-change) and humanitarian (Schwartz's universalism) vs. self-enhancement values.

Based on rigorous empirical research, the framework of values described by Schwartz maps reasonably well onto the framework of values used by policymakers. For example, the ten school values derived from the Australian Government's 'Values Educations Study' include: Social Justice (*pursuit and protection of the common good*) and Excellence (*seeking to accomplish something noteworthy and admirable*). These are compatible with Schwartz's universalism (humanitarian) and achievement (self-enhancement) values (see additional file 1). The congruence between policymakers, pedagogic and psychological perspectives provides further evidence that values are universally recognised [8,9] and that all three perspectives can assess similar values irrespective of the exact measurement instrument used [11].

### Values and behaviour

Among the few existing studies, there is evidence that specific values relate to a number of everyday (not exclusively health-related) behaviours in a plausible manner. In a convenience sample of undergraduates, Bard & Schwartz [10] found strong concordance between several (self-reported) behaviours such as '*observing traditions and holidays*' and the appropriate value (tradition), including some that could be considered health-risks. Achievement values were associated with stress-related behaviours (taking on too many commitments) and hedonism with over-eating. Both hedonism/stimulation and traditional values were strongly linked to aggregate measures of appropriate hedonistic or traditional behaviours (r >.62). Further, when self-reported behaviours were substituted with partner or peer ratings of participants' behaviours the association remained strong, providing further validation. While Bard and Schwartz [10] provide evidence of a link between values and very general behaviours, our focus is on specific health-compromising behaviours.

#### General substance-use/risk

Even fewer studies have examined the link between young people's values and health-related behaviours, but of these, most concentrate on substance-use. We briefly review this literature, finding strong cross-sectional associations, but also considerable methodological limitations.

One difficulty is that much research has conflated different types of risk-behaviours, sometimes creating a composite risk-score from a disparate selection of problem behaviours encompassing sexual behaviour, violence, substance-use, etc [12]. In a recent cross-sectional study of 10-12 year old school children from the Bahamas, certain values (conservative/traditional) were associated with fewer, and others (self-enhancement/achievement) with greater problem behaviours, although the latter result applied only to boys [12].

Using a composite indicator of substance-use in schoolchildren (ages 10-11) Cole *et al*. [13] found only traditional (conformity, tradition, security) and humanitarian (benevolence, universalism/equality) values were associated with lower substance-use. However, a family study of American adolescents (aged 18), found that while traditional values were again protective, humanitarian (humanitarianism and equality) values were associated with higher (general) substance-use [14]. This last result may be attributable to the over-sampling of 'unconventional families'. Garnier and Stein [15,16], in a study somewhat similar to our own but focusing on 205 conventional and unconventional families, found no relationship between a composite substance-use indicator and two composite indicators of teen (aged 18) values labelled traditional/achievement and humanitarian/egalitarian values respectively. However, it is difficult to draw conclusions in relation to specific substances-use from this and similar studies because of the aggregate outcomes used, and the unrepresentative nature of the families involved.

#### Specific substance-use

In a study of high school student's (ages 15-16) problem behaviours, achievement (being well-respected) values were associated with lower, and hedonistic (fun and enjoyment) values with higher use of three substances (smoking, alcohol, and marijuana) [17]. Additionally, higher rates of smoking and alcohol (but not marijuana) were linked to achievement values (self-respect/accomplishment).

One diary study investigating alcohol-use among college students found alcohol consumption moderately correlated with both hedonistic (*r *= 0.21-.29) and self-enhancement values (*r *= 0.36) values, but negatively correlated with humanistic (universalism and benevolence, r = -0.22-.25) values [18]. Additionally, traditional (tradition, conformity) values were associated with reduced problem-drinking.

Few studies have focused exclusively on smoking and values. In a random sample of UK adults, heavy-smokers held stronger achievement and hedonistic (social recognition, exciting life) values while light-smokers held more traditional and humanistic (family/national security, salvation, inner harmony) values [19]. A replication with Canadian students found that while non-smokers possessed greater humanistic (wisdom and beauty) values, smokers prioritised self-direction and self-enhancement (freedom, broadmindedness, independence, social recognition) values [20].

More recently, a study by Chen [21] examined 'terminal' and 'instrumental' values and smoking in young Chinese medical students, reporting that both were associated with smoking. Terminal values are those focused on end-goals, thus seeking either a comfortable or an exciting life are both terminal values. In contrast, instrumental values focus on conduct, e.g. valuing independence or obedience. The study's strength was that it controlled for relevant confounds (age, gender, race, sensation seeking), but because it focused on the function (terminal vs. instrumental) rather than specific types (e.g. traditional vs. anti-authority) of values, comparison with the larger literature is precluded.

### Summary

In summary, although the evidence-base is limited (often restricted to student populations), a consistent finding is that traditional values are protective, while self-direction (independence) values are risk factors for substance-use. Self-enhancement (ambition) values can be protective or not dependent upon the context, age group or substance, e.g. they are potentially a risk factor in environments with a work-hard, play-hard ethos such as college. Humanitarian values are also inconsistently associated with substance-use, possibly because of the association between cannabis-use and peace-activist/humanitarian movements.

However, the evidence-base remains inadequate for at least two reasons. Firstly, only a few [12,21] studies have adjusted for relevant confounds such as gender, parental substance-use, family structure, religion, social background or school. In addition, although sensation or risk-seeking is strongly associated with substance-use, few studies have examined its impact on the association between values and substance-use [12,21]. Secondly, and most importantly, no study has adjusted for prior substance-use; in the absence of longitudinal studies, it is impossible to evaluate how well values predict future substance-use.

#### Aims

Using a large representative longitudinal sample of young people, surveyed at ages fifteen and again at 18/19, we have three aims; first, to replicate previous cross-sectional studies finding associations between values and smoking, alcohol-use and drug-use; second, to determine if these associations remain after adjustment for relevant confounds; third, to establish if values predict future substance-use at age 18/19, after adjusting for both confounds and past health-behaviour.

## Methods

The material for the study is drawn from a Scottish longitudinal community health and lifestyle survey of young people, administered first in-school via questionnaire (ages 11, 13, and 15) and then in the post-education period by nurse interview at age 18/19. The focus here is on data collected between 1999 (aged 15) and 2003 (age 18/19) within the framework of the 'West of Scotland 11 to 16 Study/16+' [22]. The study received approval from Glasgow University's Ethics Committee, participating Education Authorities and schools, and informed consent was obtained from the parents of all participants via 'opt-out' consent forms at ages 11, 13 and 15, verbal consent from participants at each wave and written consent at age 18/19.

Due to the school-based nature of the sample the sampling scheme involved several elements to ensure a representative sample at both the primary and secondary school stages and sufficient school units to investigate or control for school-level clustering [23]. Briefly, the survey used a reverse sampling procedure which randomly selected 43 secondary schools stratified by religious denomination and deprivation, with a separate stratum for independent vs. local authority run schools. These 43 secondary schools were used to select a random sample of 135 primary schools, comprising 'feeder schools', together with those making a high number of placing requests. From these primary schools, classes were randomly selected with all pupils in the classes eligible to participate. Of the 2793 pupils who attended the 43 targeted secondary schools, 2586 (93%) participated in the baseline (age 11) survey. At age 13, the number of participants reduced to 2371 (85%), and by 15 to 2196 (79%), as expected losses in the post-school period substantially reducing the sample size at age 18/19 to 1256 (45%). Full details of the sampling strategy are available elsewhere [23].

At age 11 the sample was representative (in terms of sex and social class composition) of 11 year olds in the study area [24]. Differential attrition made later waves less representative, with attrition greater among lower social class groups, school truants, pupils of lower ability and with greater emotional and behavioural problems. To compensate for these biases, a weighting scheme was derived [24]. Use of these weights did not substantively alter any of the results presented here. The data used in this paper refer to 2196 pupils in their final year of compulsory education in 43 mainstream secondary schools in the Glasgow area, 1256 of whom provided information when aged 18/19. Parents provided information on pupils' religious background and family socioeconomic status via a supplementary questionnaire in the first wave (age 11) of the study.

### Measures

In 1999 (aged 15) pupils were asked to rate on a 5-point Likert scale (strongly agree to strongly disagree) how much they endorsed 32 questions relating to values and social attitudes, derived from a number of well-established studies of young people's values [25-28] (see additional file 2). Principal components analysis (varimax) reduced the 32 items to eight factors accounting for 45% of the variance (see additional file 1). The eight factors are broadly comparable with the generic values found by other researchers and each can be located within Schwartz's circumplex model (see additional file 1), and are labeled as follows; Traditional sex-roles (Schwartz's Tradition), e.g. '*Some equality in marriage is a good thing but by and large the husband ought to have the main say*'; Work Ethic (Schwartz's Conformity: self-discipline), e.g. '*Even if I didn't like the work, I would still want to do it as well as I could*'; Equity (Schwartz's Universalism: equality), e.g. '*The government should tax the rich more in order to help the poor*'; Citizenship and sense of belonging (Schwartz's Security: sense of belonging), e.g. '*It is a privilege to be Scottish*'; Anti-authority (Schwartz's *Anti-*tradition: no respect for tradition/obedience), e.g. '*Young people today don't have enough respect for traditional values*' (reverse scored); *Anti-*traditional - apolitical or environmentalist - politics (Schwartz's Universalism/*Anti*-Power: protect environment), e.g. "*There should be restrictions on car drivers in the city to cut down on pollution*'; Materialism (Schwartz's Power/Achievement: wealth, successful), e.g. '*There's nothing wrong with having a big house or an expensive car*'; Individualism (Schwartz's Power/*Anti*-Universalism: social power, social justice), e.g. '*The idea that society owes you a living is out of date*'. The vast majority of these items have been validated in past studies, but they do not originate from established values scales. However, given the universal nature of values and the high face validity of many items it is highly likely our items are strongly correlated with equivalent items drawn from an established values scale e.g. "*Parents can tell you what to do*" (our item) Vs "*Honoring parents*" (equivalent item Schwartz values scale; see figure 1 and additional file 1).

Several background factors related to either values or substance-use at age 15 were recorded. An area deprivation score, range 1 (least) to 7 (most deprived), was derived from pupils' postal codes using the 'Carstairs' [29] index, a standard measure based upon census data. Social class of the head of household was derived from parental questionnaires completed at wave one (age 11), coded using the standard UK classification system [30] and categorized as non-manual, manual, or missing. Religious affiliation was obtained from parents and categorized, Church of Scotland (Protestant), Catholic, Muslim, other (Jewish, Methodist, Baptist, etc) and 'none, atheist/agnostic'. At age 15, pupils family structure was coded as 2-parent, 1-parent, reconstituted (one 'birth' parent and new partner) or other (relative, foster parent, or other carer). Principal component (varimax) analysis of the (age 15) 6-item Brief Parental Bonding Instrument [31], produced two scales representing (low) parental care, e.g. 'My parents help me as much as I need' (reversed) and (high) control, e.g. 'My parents treat me like a baby'. At age 15, pupils reported parental smoking status which was used as a proxy for parental substance-use. At age 15, a generic 4-point Likert (very true to very untrue) scale asked pupils if they identified as a 'risk-taker' and is arguably a good proxy for stimulation and hedonistic (desiring an exciting/varied life) values. Due to low cell frequencies very untrue and untrue categories were collapsed for analysis.

With respect to measures of substance-use at ages 15 and 18/19, smoking was defined as regular smoking, derived from a 5-point frequency scale (never smoked, tried, used to, occasionally or regularly smoke) and is similar to that used by the UK Office for National Statistics [32]. Alcohol-use was assessed on a 7-point frequency scale ('every day' to 'I never had an alcoholic drink') and dichotomized into frequent (drink most days) vs. less frequent use. Pupils reported using a variety of illegal (primarily marijuana) drugs, dichotomized into weekly drug-use vs. less frequent use. In order to assess if our results also applied to moderate levels of substance-use, an additional set of indicators with a lower threshold were chosen for smoking (regular or occasional), alcohol (drank weekly) and drug-use (ever used). Since the results of both sets of analyses were very similar, we elected to present the more severe outcomes because of their greater relevance to public health. Those relating to more moderate outcomes are available upon request.

### Statistical analysis

The analysis used logistic regression to determine the association between values at age 15 and both concurrent and later (age 18/19) substance-use. Analysis was conducted first unadjusted and then adjusted for background factors and included past substance-use for age 18/19 outcomes. We constructed weights to compensate for differential attrition (21), but use of these weights did not alter results, nor did adjusting for school clustering (either via multilevel modeling or adjusting estimates for clustering). The influence of missing data was further explored by comparing results for models using three different methods for dealing with missing data; complete data only; including an additional missing data category for variables with more than modest amounts (50+ cases) of missing data and multiple imputation methods. Multiple imputation was implemented using the STATA 'ice' procedure and included all variables from the relevant model. Categorical variables were imputed using logit or multiple logit, continuous variables using regression and deprivation using ordinal logit commands. Ten imputed datasets were used to calculate the final combined estimates. Although the results for each method were not substantively different, we report results based on multiple imputation. Results from all other alternative models are available upon request.

## Results

### Univariate results

Table 1 reports descriptive statistics of each of the variables, including the number of cases with missing data for each variable. The substance-use outcomes display the typical pattern expected between ages 15-18/19, showing increased use of all three substances. However, the increase is most noticeable for alcohol-use.

**Table 1:** Frequencies of categorical predictors at age 15 and outcomes at age 15 and 18/19 variables.

Categorical variables	N	Frequency (%)
Sex (miss = 0)		
Female	1080	49.18
Male	1116	50.82
Parental Smoking (miss = 6)		
No	547	24.98
No parent figure	32	1.46
Yes	1,611	73.56
Social Class (miss = 161)		
Manual	1,069	52.53
Non-manual	966	47.47
Family structure (miss = 0)		
2-parent	1571	71.54
1-parent,	351	15.98
reconstituted	224	10.20
other	50	2.28
Religion (miss = 277)		
Protestant	813	42.37
Roman Catholic	653	34.03
Muslim/Islam	48	2.50
Other (Baptist, Jewish, etc)	112	5.84
None/Atheist/Agnostic	293	15.27
Risk taking (miss = 11)		
Very true	205	9.38
True	1,188	54.37
Untrue or very untrue	792	36.25

**Substance-use outcomes (moderate)**		

Smoking*, age 15 (miss = 2)	559	25.48
Smoking*, age 18/19 (miss = 980)	350	28.78
Weekly alcohol, age 15 (miss = 8)	547	25.00
Weekly alcohol, age 18/19 (miss = 980)	570	46.88
Illegal drugs, ever, age 15 (miss = 5)	882	40.26
Illegal drugs, ever, age 18/19 (miss = 980)	679	55.84

**Substance-use outcomes (severe)**		

Regularly smoke**, age 15 (miss = 2)	481	21.92
Regularly smoke**, age 18/19 (miss = 980)	291	23.93
Drink most days, age 15 (miss = 8)	69	3.15
Drink most days, age 18/19 (miss = 980)	70	5.76
Illegal drugs, weekly use, age 15 (miss = 5)	265	12.09
Illegal drugs, weekly use, age 18/19 (miss = 980)	154	12.66

*Occasional or regular smoking; ** regular smoking

miss = missing values

Note: 94 cases had missing values on the eight values factors, 145 on the area deprivation score and 1 case on the low parental care and control scales.

### Cross-sectional results

Table 2 shows the contemporaneous associations between values and substance-use among 15-year olds. All but two values (citizenship, a key value for policymakers and materialism) significantly predict at least one type of substance-use at age 15, although the effect size varies from small to moderate. Remarkably, there is only modest attenuation when nine background variables are included in our models.

**Table 2:** Cross-sectional associations between values and regular substance-use at age 15, unadjusted and mutually adjusted odds-ratios.

	Regular smoker		Frequent (most days) drinker		Weekly drug-use	
			
Predictors	Unadjusted	Adjusted	Unadjusted	Adjusted	Unadjusted	Adjusted
Traditional						
(Trad) Sex-Roles	1.09 (0.96-1.23)	**1.22 (1.05-1.41)**	**1.60 (1.24-2.07)**	**1.40 (1.09-1.81)**	**1.67 (1.45-1.92)**	**1.53 (1.30-1.81)**
(Pro) Work Ethic	**1.17 (1.03-1.32)**	**1.18 (1.04-1.33)**	1.13 (0.84-1.51)	1.10 (0.85-1.44)	1.11 (0.96-1.28)	1.06 (0.91-1.24)
(Pro) Citizenship	1.04 (0.93-1.16)	1.06 (0.94-1.20)	1.09 (0.83-1.44)	1.14 (0.88-1.48)	1.03 (0.89-1.19)	1.01 (0.87-1.18)
Humanitarian						
(Pro) Equity	**1.27 (1.15-1.42)**	**1.25 (1.12-1.39)**	0.97 (0.77-1.21)	0.99 (0.78-1.24)	1.13 (0.97-1.30)	1.09 (0.93-1.27)
Self-direction						
(Anti) Authority	**1.53 (1.35-1.74)**	**1.33 (1.17-1.51)**	**1.67 (1.31-2.13)**	1.33 (0.98-1.79)	**1.64 (1.40-1.91)**	**1.45 (1.21-1.75)**
(Anti-trad) Politics	**1.49 (1.35-1.64)**	**1.39 (1.23-1.57)**	**1.41 (1.08-1.83)**	**1.38 (1.02-1.77)**	**1.48 (1.31-1.67)**	**1.32 (1.17-1.49)**
Self-enhancement						
(Pro) Materialism	0.99 (0.88-1.11)	0.96 (0.85-1.09)	1.08 (0.78-1.48)	0.99 (0.72-1.36)	1.11 (0.94-1.31)	1.04 (0.87-1.23)
(Pro) Individualism	**0.83 (0.72-0.94)**	**0.84 (0.73-0.96)**	0.88 (0.66-1.17)	0.86 (0.64-1.14)	**0.77 (0.65-0.91)**	**0.78 (0.66-0.92)**

Parental Smoking						
No	1.00	1.00	1.00	1.00	1.00	1.00
No parent figure	2.20 (0.95-5.10)	1.27 (0.35-4.64)	**5.78 (1.86-17.93)**	2.24 (0.19-27.21)	**3.36 (1.63-6.94)**	2.03 (0.55-7.56)
Yes	**1.73 (1.40-2.15)**	**1.36 **(**1.04-1.76)**	0.82 (0.44-1.53)	0.91 (0.45-1.87)	**1.64 (1.25-2.15)**	**1.40 (1.00-1.96)**

Risk taking						
Very true	**5.25 (3.75-7.36)**	**4.25 (2.89-6.26)**	**10.01 (4.95-20.23)**	**4.26 (1.84-9.85)**	**10.98 (6.66-18.11)**	**5.94 (3.52-10.03)**
True	**2.74 (2.16-3.48)**	**2.33 (1.79-3.04)**	**2.95 (1.27-6.86)**	1.87 (0.74-4.69)	**4.12 (2.83-6.00)**	**2.81 (1.90-4.16)**
Untrue/v untrue	1.00	1.00	1.00	1.00	1.00	1.00

Sex						
Female	1.00	1.00	1.00	1.00	1.00	1.00
Male	**0.73 (0.58-0.93)**	**0.50 (0.37-0.66)**	**2.84 (1.56-5.14)**	1.82 (0.97-3.40)	**1.90 (1.45-2.49)**	1.10 (0.80-1.52)
						
Area deprivation	1.02 (0.96-1.10)	**0.93 (0.86-1.00)**	**0.88 (0.78-0.99)**	**0.85 (0.75-0.96)**	**1.09 (1.01-1.19)**	1.02 (0.93-1.13)
						
Social Class						
Manual	1.00	1.00	1.00	1.00	1.00	1.00
Non-manual	**0.72 (0.56-0.93)**	0.86 (0.67-1.09)	1.02 (0.67-1.54)	1.05 (0.66-1.67)	0.65 (0.48-0.88)	0.84 (0.57-1.23)
						
Family structure						
2-parent	1.00	1.00	1.00	1.00	1.00	1.00
1-parent	**2.19 (1.65-2.91)**	**1.78 (1.29-2.45)**	0.91 (0.49-1.69)	0.79 (0.37-1.67)	**1.88 (1.42-2.49)**	**1.41 (1.03-1.93)**
reconstituted	**2.07 (1.47-2.93)**	**1.71 (1.18-2.50)**	1.49 (0.72-3.05)	1.42 (0.64-3.16)	**1.86 (1.24-2.78)**	**1.60 (1.07-2.40)**
other	**1.95 (1.04-3.66)**	1.19 (0.46-3.05)	**5.65 (2.17-14.71)**	3.34 (0.44-25.27)	**2.88 (1.42-5.82)**	1.46 (0.49-4.36)
						
Low parental care	**1.24 (1.15-1.33)**	**1.11 (1.04-1.19)**	**1.34 (1.17-1.53)**	**1.19 (1.00-1.40)**	**1.22 (1.16-1.29)**	1.06 (0.98-1.15)
Parental control	1.01 (0.91-1.11)	0.96 (0.86-1.08)	1.11 (0.91-1.36)	1.03 (0.83-1.28)	1.13 (0.99-1.28)	1.07 (0.95-1.21)
						
Religion						
Protestant	1.00	1.00	1.00	1.00	1.00	1.00
Catholic	**0.75 (0.56-0.99)**	0.76 (0.55-1.04)	1.42 (0.68-2.95)	1.67 (0.80-3.46)	1.21 (0.80-1.81)	1.24 (0.81-1.90)
Muslim/Islam	0.47 (0.15-1.42)	0.62 (0.18-2.19)	0.00 (none) †	0.00 (none) †	0.35 (0.07-1.69)	0.32 (0.06-1.68)
Other	**0.59 (0.35-1.00)**	0.79 (0.48-1.29)	1.20 (0.34-4.26)	1.18 (0.31-4.44)	1.01 (0.54-1.91)	1.40 (0.68-2.87)
None or atheist	0.92 (0.63-1.33)	0.76 (0.52-1.12)	2.23 (0.89-5.55)	1.97 (0.76-5.11)	1.16 (0.76-1.78)	1.00 (0.65-1.55)

Significant odds ratios (p = 0.05 or lower) are shown in bold.

† = No Muslim participant reported drinking alcohol; accordingly the final model excluded Muslim participants.

With the single exception of alcohol-use, both anti-authority and anti-traditional politics predicted increased substance-use of any type, with anti-authority typically the strongest predictor. Traditional sex-roles also predicted increased substance-use irrespective of type, although only in the adjusted model in relation to smoking. Work ethic was associated with increased regular smoking, individualism with lower smoking and drug-use and equity with a significant, but small, increase in smoking.

### Longitudinal results

Table 3 presents the longitudinal results, testing how well values at age 15 predict substance-use at age 18/19, unadjusted and adjusted for background factors. Although most control variables remain significant, prior (age 15) substance-use is unsurprisingly the strongest predictor of substance-use at age 18/19. There are a few significant unadjusted associations between values and future substance-use, which are broadly compatible with the cross-sectional results. Thus anti-authority values consistently predict increased smoking and drug-use; traditional sex-role values, predict increases in each substance-use at age 18/19; anti-traditional politics and work ethic values again predict increased smoking. However, only two associations, that between work ethic and regular smoking and anti-authority and regular drug-use, remained (marginally) significant when adjusted for background factors and past substance-use. Associations between values and the more moderate substance-use outcomes were even weaker; in the final adjusted longitudinal model not a single association was significant.

**Table 3:** Longitudinal associations between values and regular substance-use behaviour at age 18/19, unadjusted and mutually adjusted odds-ratios

	Regular smoker		Frequent (most days) drinker		Weekly drug-use	
			
Predictors	Unadjusted	Adjusted	Unadjusted	Adjusted	Unadjusted	Adjusted
Traditional						
(Trad) Sex-Roles	**1.19 (1.04-1.37)**	1.19 (0.98-1.46)	**1.30 (1.02-1.65)**	1.03 (0.73-1.44)	**1.62 (1.33-1.99)**	1.10 (0.88-1.38)
(Pro) Work Ethic	**1.24 (1.10-1.41)**	**1.21 (1.02-1.45)**	1.02 (0.78-1.32)	0.99 (0.77-1.27)	1.11 (0.95-1.31)	1.00 (0.85-1.18)
(Pro) Citizenship	1.04 (0.91-1.21)	1.06 (0.88-1.27)	0.98 (0.77-1.25)	0.95 (0.74-1.23)	1.11 (0.96-1.29)	1.03 (0.88-1.21)
Humanitarian						
(Pro) Equity	1.12 (0.96-1.30)	0.95 (0.77-1.18)	1.05 (0.74-1.48)	1.10 (0.80-1.52)	1.10 (0.92-1.31)	1.07 (0.88-1.31)
Self-direction						
(Anti) Authority	**1.21 (1.05-1.39)**	0.92 (0.76-1.11)	1.20 (0.89-1.61)	1.05 (0.78-1.42)	**1.52 (1.29-1.79)**	**1.33 (1.10-1.61)**
(Anti-trad) Politics	**1.38 (1.21-1.59)**	1.15 (0.96-1.38)	0.85 (0.66-1.08)	0.83 (0.64-1.09)	**1.15 (1.00-1.33)**	0.97 (0.84-1.12)
Self-enhancement						
(Pro) Materialism	1.02 (0.86-1.20)	1.01 (0.80-1.29)	1.22 (0.97-1.54)	1.13 (0.89-1.44)	1.01 (0.84-1.22)	0.92 (0.74-1.13)
(Pro) Individualism	0.99 (0.84-1.17)	1.14 (0.93-1.41)	1.14 (0.87-1.51)	1.13 (0.85-1.49)	0.99 (0.84-1.18)	1.06 (0.88-1.27)

Prior subst-use *						
None	1.00	1.00	1.00	1.00	1.00	1.00
Regular prior- use	**17.14 (11.88-24.71)**	**16.76 (11.63-24.16)**	**3.27 (1.03-10.38)**	2.38 (0.70-8.04)	**7.96 (5.51-11.48)**	**5.52 (3.60-8.48)**
						
Parental Smoking						
No	1.00	1.00	1.00	1.00	1.00	1.00
No parent figure	**3.46 (1.25-9.57)**	1.74 (0.20-14.91)	†	†	1.62 (0.44-5.92)	0.52 (0.03-7.74)
Yes	**1.68 (1.19-2.39)**	1.23 (0.75-2.03)	0.67 (0.34-1.32)	0.79 (0.37-1.72)	1.32 (0.91-1.92)	1.20 (0.72-2.00)

Risk taking						
Very true	**3.38 (2.29-5.01)**	1.37 (0.80-2.33)	**3.39 (1.66-6.93)**	**2.39 (1.06-5.38)**	**7.28 (3.92-13.50)**	**3.31 (1.65-6.62)**
True	**2.08 (1.56-2.77)**	1.29 (0.90-1.86)	**1.79 (1.02-3.12)**	1.66 (0.91-3.01)	**3.20 (2.01-5.07)**	**2.09 (1.26-3.46)**
Untrue/v untrue	1.00	1.00	1.00	1.00	1.00	1.00

Sex						
Female	1.00	1.00	1.00	1.00	1.00	1.00
Male	0.98 (0.76-1.28)	0.93 (0.61-1.43)	**2.54 (1.46-4.41)**	**2.04 (1.04-4.00)**	**3.54 (2.26-5.54)**	**2.97 (1.86-4.75)**
						
Area deprivation	**1.08 (1.00-1.17)**	1.03 (0.92-1.16)	0.98 (0.81-1.18)	1.11 (0.89-1.38)	1.09 (0.97-1.21)	1.08 (0.95-1.22)
						
Social Class						
Manual	1.00	1.00	1.00	1.00	1.00	1.00
Non-manual	**0.63 (0.47-0.85)**	0.77 (0.48-1.21)	**2.04 (1.12-3.70)**	**2.20 (1.15-4.21)**	0.79 (0.54-1.13)	0.98 (0.66-1.46)
						
Family structure						
2-parent	1.00	1.00	1.00	1.00	1.00	1.00
1-parent	**1.75 (1.21-2.52)**	1.10 (0.68-1.78)	0.82 (0.40-1.68)	0.80 (0.36-1.78)	1.12 (0.78-1.62)	0.81 (0.49-1.35)
reconstituted	**2.26 (1.55-3.30)**	**1.78 (1.15-2.78)**	0.58 (0.21-1.60)	0.55 (0.16-1.89)	1.27 (0.73-2.20)	1.09 (0.58-2.08)
other	**2.95 (1.43-6.09)**	1.92 (0.37-9.89)	†	†	2.05 (0.79-5.31)	1.95 (0.27-14.16)
						
Low parental care	**1.16 (1.06-1.26)**	1.05 (0.92-1.19)	1.15 (1.02-1.29)	1.11 (0.97-1.28)	**1.17 (1.07-1.27)**	1.04 (0.92-1.16)
Parental control	1.01 (0.90-1.13)	0.98 (0.85-1.13)	0.98 (0.79-1.20)	0.93 (0.76-1.15)	1.01 (0.89-1.15)	0.93 (0.80-1.07)
						
Religion						
Protestant	1.00	1.00	1.00	1.00	1.00	1.00
Catholic	0.88 (0.65-1.20)	1.03 (0.72-1.48)	1.07 (0.63-1.79)	1.10 (0.64-1.87)	0.73 (0.52-1.03)	**0.67 (0.46-0.97)**
Muslim/Islam	0.93 (0.41-2.14)	1.54 (0.66-3.62)	0.00 (none) ‡	0.00 (none) ‡	0.52 (0.15-1.74)	0.51 (0.14-1.86)
Other	0.64 (0.35-1.20)	0.92 (0.41-2.08)	1.01 (0.33-3.03)	0.80 (0.26-2.49)	0.68 (0.33-1.39)	0.67 (0.29-1.57)
None or Atheist	1.05 (0.65-1.71)	1.24 (0.67-2.30)	1.68 (0.90-3.12)	1.51 (0.83-2.74)	0.77 (0.49-1.21)	0.61 (0.39-0.97)

Significant odds ratios (p = 0.05 or lower) are shown in bold.

* = Prior use refers to use of the same substance as the outcome, e.g. for the smoking at age 18/19 outcome this means smoking at age 15.

† = Due to low cell frequencies this category been collapsed into another (adjacent) grouping.

‡ = No Muslim participant reported drinking alcohol; accordingly the final model excluded Muslim participants.

## Discussion

Returning to our three aims, we replicated the well-established cross-sectional links between values and substance-use found in the literature. Secondly, we established that background factors did not explain these cross-sectional associations. Finally, but most importantly, although some values do predict future substance-use in simple (unadjusted) analyses, after adjusting for background and prior substance-use almost all the associations either vanish or are reduced to marginal effect sizes.

### Cross-sectional associations

With respect to the cross-sectional findings, the associations between specific values and substance-use in this study are compatible with past research, but intriguingly not all values typically thought to reduce substance-use did so. This raises questions about what values are 'good' in relation to substance-use. As expected, independence and rebellious orientated (anti-authority, non-traditional or apolitical) values were associated with greater substance use. However, several unexpected associations are more notable, but require explanation. The links between traditional sex-roles and increased substance-use and between work ethic and smoking, suggests that certain cultures, competitive environments, or 'masculine' sex-roles [33] encourage substance-use. Substance-use may be a coping strategy linked to the additional stress associated with work-orientated values and is compatible with research demonstrating that achievement values are linked to certain stress inducing behaviours, i.e. "taking on too many commitments" [10]. The adoption of stereotypically 'masculine' forms of coping and bonding behaviours, such as heavy-drinking or cannabis-use in response to excessive workloads, could explain this link. Equity values also predicted increased smoking rates, but only marginally. A surprising finding is that individualism (speculated to be associated with negative [34] outcomes) was linked to reduced substance-use; in fact, more individualistic young people had lower levels of smoking and drug use.

### Longitudinal associations

Our longitudinal findings directly challenge the proposition that authoritarian or traditional values are necessarily better for health than liberal or individualist ideals [1-3]. However, since our study only focused on substance-use, it is possible that individualistic values predict other health and health-related outcomes such as poorer mental health. Furthermore, we did not measure values at both time points, our underlying assumption being that values are relatively stable from mid-adolescence onward (see additional file 1). This assumption has some support, but we cannot eliminate the possibility that values changed between ages 15 and 18/19. Nonetheless, this does not weaken the argument that (based on our results) moral values have poor predictive power in relation to later substance-use from mid to late-adolescence.

From a developmental perspective it is of course possible that values in childhood or early adolescence may predict uptake before age 15, but given the consensus that values are not fully formed until mid to late adolescence [35-37] this may be a difficult (although not impossible) research question to test. A strong association between substance-use and values before age 15 may leave little variation to explain in the post-school period. However, the large increase in alcohol-use between each wave suggests there is substantial change between ages 15 and 18/19. Finally, compatible with the literature on values transmission (see additional file 1) we found that background factors explained very little of the cross-sectional associations between values and substance-use. This suggests that the typical structural explanations for this link (e.g. religion or class) are of minor relevance during this life-stage, although there always remains the possibility of model misspecification.

### Policy considerations

Should policymakers encourage certain moral values on the assumption that this will inhibit later (early adult) substance-use? The evidence from our study suggests not, or at least not to any significant degree. If the aim is to promote values deemed socially desirable, then this is a wider moral question largely detached from public-health policy. Advocates of values education will likely focus on the cross-sectional results and the two (marginally) significant longitudinal associations between anti-authority/political values and substance-use, while their opponents will likely concentrate on the small effect size(s) and the, generally, negative longitudinal findings. We do not wish to discourage innovative approaches to reducing population levels of substance-use among young people, but it is clear from our results that future values-based studies and interventions should be rigorous (i.e., a prospective study, which adjusts for relevant background factors) and focused on 'practical' significance.

## Conclusion

Arguably, most people intuitively consider certain values (e.g. benevolence, equality) to be of greater ethical and social 'worth' and these values are to be encouraged. However, expanding this to encompass improved health and health-related behaviours is problematic. Our evidence suggests that these values are not necessarily 'good' (or 'bad') for you, at least in relation to substance-use.

## Competing interests

All authors are supported financially by the Medical Research Council of Great Britain as part of the Youth and Health Programme (WBS U.1300.00.007) and have no other competing interests.

## Authors' contributions

RY wrote the majority of the manuscript, conceived the theoretical approach taken in the paper, participated in design of the final phase of the 11-16/16+ study, and performed the statistical analysis. PW conceived of the original 11-16 study, and participated in its design and coordination and helped to draft the manuscript. Both authors read and approved the final manuscript.

## Pre-publication history

The pre-publication history for this paper can be accessed here:

http://www.biomedcentral.com/1471-2458/10/165/prepub

## Supplementary Material

Additional file 1 **Supplementary supporting material**. Contains 2 figures; figure 1a locates specific items within the Schwartz value model; figure 1b provides a simplified model of Schwartz's circumplex model. Box 1 contains a broad introduction to the Schwartz model and a summary of its 10 values and typical items used in their measurement. Table 1 shows the factor loadings of the 32 items after varimax factor analysis in relation to an 8 factor solution. Click here for file

Additional file 2 **Questionnaire items**. List of 32 items on opinions and beliefs used in the factor analysis. Click here for file
