# Supplementary material for: Do 'good values' lead to 'good' health-behaviours? Longitudinal associations between young people's values and later substance-use
Source: BMC Public Health. 2010 Mar 26;10:165. doi: 10.1186/1471-2458-10-165 (PMC2864210; doi:10.1186/1471-2458-10-165)
Supplement: Additional file 1 — Supplementary supporting material. Contains 2 figures; figure 1a locates specific items within the Schwartz value model; figure 1b provides a simplified model of Schwartz's circumplex model. Box 1 contains a broad introduction to the Schwartz model and a summary of its 10 values and typical items used in their measurement. Table 1 shows the factor loadings of the 32 items after varimax factor analysis in relation to an 8 factor solution. [file 1471-2458-10-165-S1.DOC]

**The structure of values (Schwartz 10-value model)**

**Figure 1a**

**
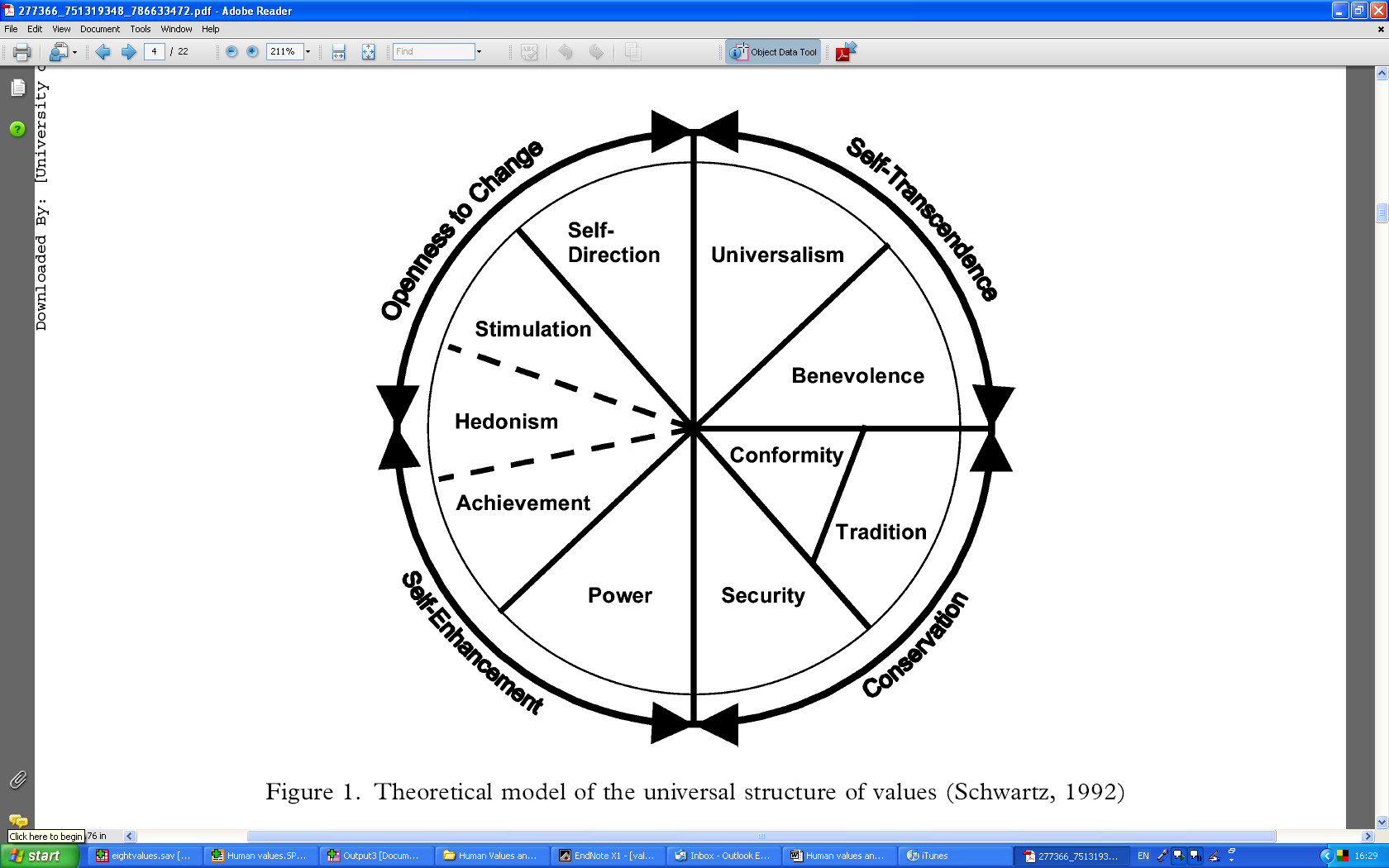
**

**Figure 1b**

**The structure of values (Schwartz 10-value model)**

**Outline of values: function, features structure and content of values**

| **Value type and Definition** | **Typical item** |
| --- | --- |
| **Benevolence:** preservation and enhancement of the welfare of people with whom one is in frequent personal contact. | honest, forgiving, loyal, spiritual life, helpful, responsible, meaning in life, true friendship, mature love |
| **Universalism:** Understanding, appreciation, tolerance, and protection for the welfare of *all* people and for nature. | inner harmony, social justice, world at peace, protect environment, equality, broad minded, unity with nature, world of beauty, wisdom |
| **Self-direction:** independent thought and action – choosing, creating exploring. | self respect, choosing own goals, creativity, curious, freedom, independent |
| **Stimulation:** excitement, novelty, and challenge in life. | exciting life, varied life, daring |
| **Hedonism:** pleasure and sensuous gratification for oneself. | pleasure, enjoying life |
| **Achievement:** personal success through demonstrating competence according to social standards. | ambitious, successful, capable, intelligent, influential |
| **Power:** social status and prestige, control or dominance over people and resources. | preserving public image, social recognition, authority, wealth, social power |
| **Security:** safety, harmony, and stability of society, of relationships, and of self. | national security, sense of belonging, reciprocation of favors, clean, social order, family security, healthy |
| **Conformity:** restraint of actions, inclinations, and impulses likely to upset or harm others and violate social expectations or norms. | obedient, honor elders, politeness, self discipline |
| **Tradition:** respect, commitment, and acceptance of the customs and ideas that traditional culture or religion provide. | accepting my portion in life, moderate, devout, detachment, respect for tradition, humble |

**Box 1: The structure of values (Schwartz 10-value model)**

**(Schwartz model)**

**Values as important psychological constructs**

Recently, interest in values as a key social and psychological construct, with the potential to predict behaviour [1, 2], has shown resurgence [2-5]. Kluckhohn [6] defines values as *"desirable goals, varying in importance, that serve as guiding principles in people's lives."*.

**Some important features of values**

Values have important features; they are believed to become relatively ‘fixed’ in early adulthood [3, 4, 7] and are therefore stable over time [5], are related to, but distinct from, personality and norms [8, 9], they are universal [5, 10], i.e. relatively invariant across nations, and (provided researchers use a wide spectrum of items) produce similar values with broadly similar structures irrespective of the measurement instrument used [5].

**The structure and content of values**

We briefly outline one well-developed value theory, Schwartz’s circumplex model [1, 11]. Using both multidimensional scaling and factor analysis, Schwartz analysed a large battery of individual questionnaire items related to values (See table opposite for typical items). Figure 1a outlines the relations between items in two-dimensional space. From this, a recognisable cluster of 10 values emerged (see table opposite for description). Figure 1b is a simplified version of the results, which display a distinct cirumplex structure, implying certain values are less likely to be associated with each other. Further, figure 1b groups the values into four polarised higher-order more generic values.

**Where do people acquire their values?**

It remains unclear how exactly values are established, but it seems logical to presume familial or intergenerational transmission of values is likely. However, studies of familial transmission of values find surprisingly low correlations between parent’s and children’s values [12], suggesting other factors such as media [13], consumerism [14, 15], peers and significant others [16, 17] or school [18] may play a role.

**Table1: Factor analysis of 32 values and opinions items**

| Questionnaire item | Sex  Role | Work  Ethic | Equity | Authority | Anti-trad politics | Citizen-ship | Material-ism | Individual-ism |
| --- | --- | --- | --- | --- | --- | --- | --- | --- |
| Husband has main say | .76 |  |  |  |  |  |  |  |
| Men to earn, women to care | .73 |  |  |  |  |  |  |  |
| Equal male-female work opposed | -.72 |  |  |  |  |  |  |  |
| Gender equality in house | -.71 |  |  |  |  |  |  |  |
| Women should care for relations | .66 |  |  |  |  |  |  |  |
| Job to feel member society | .33 |  |  |  |  |  |  | .31 |
| Pack in disliked job |  | -.70 |  |  |  |  |  |  |
| Any job better then unemployed |  | .62 |  |  |  |  |  |  |
| Imp to hang onto job |  | .61 |  |  |  |  |  |  |
| Do good work |  | .48 |  |  |  |  |  |  |
| Gov tax rich more |  |  | .60 |  |  |  |  |  |
| Lower standard of living |  |  | .54 |  |  |  |  |  |
| Gov help people get local jobs |  |  | .46 |  |  |  |  |  |
| Maintain material standards |  |  | .44 |  |  |  |  |  |
| Pars can boss you |  |  |  | .61 |  |  |  |  |
| Police friendly to young people |  |  |  | .54 |  |  |  |  |
| Greatest loyalty to family |  |  |  | .53 |  | .35 |  |  |
| Church best authority |  |  |  | .47 |  |  |  |  |
| Yp not respectful enough |  |  |  | .35 |  |  |  |  |
| Possible to find a job |  |  |  | .31 |  |  |  |  |
| Xs attention to environment |  |  |  |  | .62 |  |  |  |
| Political parties all same |  |  |  |  | .54 |  |  |  |
| Restrict cars in city |  |  | .46 |  | -.47 |  |  | .32 |
| Privilege to be Scottish |  |  |  |  |  | .70 |  |  |
| Best to live here |  |  |  |  |  | .60 |  |  |
| Young people should feel/belong |  |  |  |  |  | .48 |  |  |
| Social exclusion a problem |  |  |  |  |  |  | .58 |  |
| Big house/car ok |  |  | -.36 |  |  |  | .57 |  |
| Get satisfaction without job |  |  |  |  |  |  | .56 |  |
| Personal plate good | .32 |  |  |  | .36 |  | .42 |  |
| Society doesn’t owe you |  |  |  |  |  |  |  | .62 |
| Too much dependency |  |  |  |  |  |  |  | .57 |

Principal components with varimax rotation.

Loadings below 0.3 suppressed and leading zeros omitted.

Anti-trad politics = Anti-traditional (Apolitical, or environmental) politics.

**REFERENCES**

1. Bardi A, Schwartz SH: **Values and behavior: Strength and structure of relations**. *Personality and Social Psychology Bulletin* 2003, **29**(10):1207-1220.

2. Hitlin S, Piliavin JA: **Values: Reviving a dormant concept**. *Annual Review of Sociology* 2004, **30**:359-393.

3. Hofstede G, Hofstede GJ: *Cultures and Organizations, Software of the Mind: Intercultural Cooperation and its Importance for Survival*. New York: McGraw-Hill; 2005.

4. Inglehart R: *Culture shift in advanced industrial society*. Princeton, N.J.: Princeton University Press; 1990.

5. Schwartz SH, Bardi A: **Value hierarchies across cultures - Taking a similarities perspective**. *Journal of Cross-Cultural Psychology* 2001, **32**(3):268-290.

6. Kluckhohn C: *Values and value-orientation in the theory of action: An exploration in definition and classification*. Cambridge, MA: Havard University Press; 1951.

7. Inglehart R: *Modernization and postmodernization: Cultural, economic, and political change in 43 societies*. Princeton, N.J.: Princeton University Press; 1997.

8. Aluja A, Garcia LF: **Relationships between Big Five personality factors and values**. *Social Behavior and Personality* 2004, **32**(7):619-625.

9. Bilsky W, Schwartz SH: **Values and Personality**. *European Journal of Personality* 1994, **8**(3):163-181.

10. Davidov E, Schmidt P, Schwartz SH: **Bringing values back in - The adequacy of the European Social Survey to measure values in 20 countries**. *Public Opinion Quarterly* 2008, **72**(3):420-445.

11. Schwartz SH: **Universals in the content and structure of values: theoretical advances and empirical tests in 20 countries**. In *Advances in Experimental Social Psychology. Volume 25*. Edited by Zanna M. New York: Academic Press; 1992:1-65.

12. Liu H, Yu S, Cottrell L, Lunn S, Deveaux L, Brathwaite NV, Marshall S, Li X, Stanton B: **Personal values and involvement in problem behaviors among Bahamian early adolescents: a cross-sectional study**. *BMC Public Health* 2007, **7**:135.

13. Besley JC: **Media use and human values**. *Journalism & Mass Communication Quarterly* 2008, **85**(2):311-330.

14. Kropp F, Lavack AM, Silvera DH: **Values and collective self-esteem as predictors of consumer susceptibility to interpersonal influence among university students**. *International Marketing Review* 2005, **22**(1):7-33.

15. Anne Dibley SB: **Uncovering the links between brand choice and personal values among young British and Spanish girls**. *Journal of Consumer Behaviour* 2001, **1**(1):77-93.

16. Lickona R: **Combatting Violence with Values: The Character Education Solution**. *Reprint from Law Studies* 1994, **19**(3):1-6.

17. Lickona T: *Educating for Character. How Our Schools Can Teach Respect and Responsibility*. New York: Bantum; 1992.

18. Hofmann-Towfigh N: **Do students' values change in different types of schools?** *Journal of Moral Education* 2007, **36**(4):453-473.
