# Supplementary material for: Do 'good values' lead to 'good' health-behaviours? Longitudinal associations between young people's values and later substance-use
Source: BMC Public Health. 2010 Mar 26;10:165. doi: 10.1186/1471-2458-10-165 (PMC2864210; doi:10.1186/1471-2458-10-165)
Supplement: Additional file 2 — Questionnaire items. List of 32 items on opinions and beliefs used in the factor analysis. [file 1471-2458-10-165-S2.DOC]

**YOUR OPINIONS**

**3. What do you think about ...?**  People have different opinions about a lot of things where there are no ‘right’ or ‘wrong’ answers. There are 32 opinion questions in this questionnaire. In this questionnaire students count as both young people and young adults. **So, do you agree with these or not?** Tick **one** box on each line.

**strongly strongly**

agree agree neither disagree disagree

| **^1^. the government should help people**  **to get jobs where they live rather**  **than expecting them to move to get work ............** | ❑ 1 **......**❑ 2 **......**❑ 3 **......**❑ 4 **......**❑ 5 |
| --- | --- |
| **^2^. the police are generally**  **helpful and friendly to young people .....................** | ❑ 1 **......**❑ 2 **......**❑ 3 **......**❑ 4 **......**❑ 5 |
| **^3^. people should realise that**  **their greatest loyalty is to their family ...................** | ❑ 1 **......**❑ 2 **......**❑ 3 **......**❑ 4 **......**❑ 5 |
| **^4^. people should accept a**  **lower standard of living to decrease**  **pollution and environmental problems .................** | ❑ 1 **......**❑ 2 **......**❑ 3 **......**❑ 4 **......**❑ 5 |
| **^5^. the government should tax**  **the rich more in order to help the poor .................** | ❑ 1 **......**❑ 2 **......**❑ 3 **......**❑ 4 **......**❑ 5 |
| **^6^. some equality in marriage**  **is a good thing, but by and large**  **the husband ought to have the main say ..............** | ❑ 1 **......**❑ 2 **......**❑ 3 **......**❑ 4 **......**❑ 5 |
| **^7^. it is better to live here**  **than in any other part of the country .....................** | ❑ 1 **......**❑ 2 **......**❑ 3 **......**❑ 4 **......**❑ 5 |
| **^8^. if you live with your parents**  **they can tell you what to do ...................................** | ❑ 1 **......**❑ 2 **......**❑ 3 **......**❑ 4 **......**❑ 5 |
| **^9^. people depend too much on the welfare**  **state and not enough on themselves**  **to provide for their health and welfare ..................** | ❑ 1 **......**❑ 2 **......**❑ 3 **......**❑ 4 **......**❑ 5 |
| **^10^. there’s too much attention**  **in the media to environmental issues ...................** | ❑ 1 **......**❑ 2 **......**❑ 3 **......**❑ 4 **......**❑ 5 |

**YOUR OPINIONS…**

**strongly strongly**

**agree agree neither di**sagree disagree

| **^11^. men and women should all have**  **the chance to do the same kind of work ...............** | ❑ 1 **......**❑ 2 **......**❑ 3 **......**❑ 4 **......**❑ 5 |
| --- | --- |
| **^12^. if you’re really determined**  **it is possible to find a job ........................................** | ❑ 1 **......**❑ 2 **......**❑ 3 **......**❑ 4 **......**❑ 5 |
| **^13^. young people today don’t have**  **enough respect for traditional values ...................** | ❑ 1 **......**❑ 2 **......**❑ 3 **......**❑ 4 **......**❑ 5 |
| **^14^. there should be restrictions on**  **car drivers in the city to cut down on pollution ....** | ❑ 1 **......**❑ 2 **......**❑ 3 **......**❑ 4 **......**❑ 5 |
| **^15^. a husband’s (man’s) job is to**  **earn the money; a wife’s (woman’s)**  **job is to look after the home and family ................** | ❑ 1 **......**❑ 2 **......**❑ 3 **......**❑ 4 **......**❑ 5 |
| **^16^. once you’ve got a job it’s important to**  **hang onto it even if you don’t really like it ............** | ❑ 1 **......**❑ 2 **......**❑ 3 **......**❑ 4 **......**❑ 5 |
| **^17^. the church is the best authority**  **to decide on matters of right or wrong ..................** | ❑ 1 **......**❑ 2 **......**❑ 3 **......**❑ 4 **......**❑ 5 |
| **^18^. if I didn’t like a job I’d pack it in,**  **even if there was no other job to go to ..................** | ❑ 1 **......**❑ 2 **......**❑ 3 **......**❑ 4 **......**❑ 5 |
| **^19^. it is a privilege to be Scottish ................................** | ❑ 1 **......**❑ 2 **......**❑ 3 **......**❑ 4 **......**❑ 5 |
| **^20^. women rather than men**  **should look after relatives who need care ............** | ❑ 1 **......**❑ 2 **......**❑ 3 **......**❑ 4 **......**❑ 5 |
| **^21^. it is very important for (young) people**  **to feel they belong to a particular society .............** | ❑ 1 **......**❑ 2 **......**❑ 3 **......**❑ 4 **......**❑ 5 |
| **^21^. it is very important for (young) people**  **to feel they belong to a particular society .............** | ❑ 1 **......**❑ 2 **......**❑ 3 **......**❑ 4 **......**❑ 5 |

**YOUR OPINIONS…**

**strongly strongly**

agree agree neither disagree disagree

| **^22^. it does not really make much difference**  **which political party is in power in Britain ............** | ❑ 1 **......**❑ 2 **......**❑ 3 **......**❑ 4 **......**❑ 5 |
| --- | --- |
| **^23^. the idea that society**  **owes you a living is out of date .............................** | ❑ 1 **......**❑ 2 **......**❑ 3 **......**❑ 4 **......**❑ 5 |
| **^24^. having almost any job**  **is better than being unemployed............................** | ❑ 1 **......**❑ 2 **......**❑ 3 **......**❑ 4 **......**❑ 5 |
| **^25^. having a personalised**  **number plate on your car is**  **a good way to show how well you’re doing ..........** | ❑ 1 **......**❑ 2 **......**❑ 3 **......**❑ 4 **......**❑ 5 |
| **^26^. even if I didn’t like the work,**  **I would still want to do it as well as I could ..........** | ❑ 1 **......**❑ 2 **......**❑ 3 **......**❑ 4 **......**❑ 5 |
| **^27^. a person must have a job**  **to feel a full member of society ..............................** | ❑ 1 **......**❑ 2 **......**❑ 3 **......**❑ 4 **......**❑ 5 |
| **^28^. men and women should do**  **the same jobs around the house ...........................** | ❑ 1 **......**❑ 2 **......**❑ 3 **......**❑ 4 **......**❑ 5 |
| **^29^. mankind should not try to increase**  **material standards of living anymore ....................** | ❑ 1 **......**❑ 2 **......**❑ 3 **......**❑ 4 **......**❑ 5 |
| **^30^. a person can get satisfaction**  **out of life without having a job ...............................** | ❑ 1 **......**❑ 2 **......**❑ 3 **......**❑ 4 **......**❑ 5 |
| **^31^. there’s nothing wrong in**  **owning a big house or an expensive car ..............** | ❑ 1 **......**❑ 2 **......**❑ 3 **......**❑ 4 **......**❑ 5 |
| **^32^. a lot of society’s problems are**  **caused by people feeling excluded from it ...........** | ❑ 1 **......**❑ 2 **......**❑ 3 **......**❑ 4 **......**❑ 5 |
